# Piezo channels in the intestinal tract

**DOI:** 10.3389/fphys.2024.1356317

**Published:** 2024-02-06

**Authors:** Haolong He, Jingying Zhou, Xuan Xu, Pinxi Zhou, Huan Zhong, Mi Liu

**Affiliations:** ^1^ School of Acupuncture-Moxibustion, Tuina and Rehabilitation, Hunan University of Chinese Medicine, Changsha, Hunan, China; ^2^ Key Laboratory of Acupuncture and Moxibustion Bioinformatics, Education Department of Hunan Province, Changsha, Hunan, China

**Keywords:** Piezo1, Piezo2, mechanosensitive ion channels, mechanosensation, intestinal tract

## Abstract

The intestine is the largest mechanosensitive organ in the human body whose epithelial cells, smooth muscle cells, neurons and enteroendocrine cells must sense and respond to various mechanical stimuli such as motility, distension, stretch and shear to regulate physiological processes including digestion, absorption, secretion, motility and immunity. Piezo channels are a newly discovered class of mechanosensitive ion channels consisting of two subtypes, Piezo1 and Piezo2. Piezo channels are widely expressed in the intestine and are involved in physiological and pathological processes. The present review summarizes the current research progress on the expression, function and regulation of Piezo channels in the intestine, with the aim of providing a reference for the future development of therapeutic strategies targeting Piezo channels.

## 1 Introduction

The gastrointestinal (GI) tract is one of the most mechanosensitive and active organs of the body, and its main function is to digest and absorb nutrients from food, as well as being an important part of the immune system and defense against foreign pathogens and toxins. In order to fulfill these functions, the intestine needs to be able to sense and adapt to a variety of mechanical stimuli from the internal and external environments, such as peristalsis, distension, stretching and shearing (Smid, 2009; Zhu et al., 2022a). These mechanical stimuli not only affect the structure and morphology of the intestine, but also the physiological activities of the intestine such as secretion, motility, absorption and immunity. Therefore, the intestine has a high degree of mechanosensitivity, which means that it is capable of converting mechanical forces into electrochemical signals, transmitting information within or between cells, and thus regulating the corresponding responses (Treichel et al., 2018).

Mechanosensitive ion channels (MSCs) are a type of membrane proteins capable of converting mechanical signals into electrochemical signals that are essential for maintaining normal cell and tissue function (Cox et al., 2019). A variety of mechanosensitive cell types are present in the gut, including epithelial enterochromaffin (EC) cells, intrinsic and extrinsic enteric neurons, smooth muscle cells, and Cajal mesenchymal stromal cells (ICC) (Yang et al., 2022). These cells utilize MSCs to create variable ion permeability across membranes, thereby enabling mechano-electrical coupling. MSCs are directly activated by stresses applied to the lipid bilayer or its associated non-membrane components. Furthermore, these channels exhibit various ion selectivity, voltage dependence, kinetic properties, and modulation, and play different roles in different mechanosensitive cells (Zhang et al., 2021; Yang et al., 2022). Exploring MSCs and their mechanisms of action in the GI tract is of great significance for understanding the normal physiological functions of the GI tract and the development of related diseases.

In 2010, Coste et al. (2010) first identified Piezo1 in a mouse neuroblastoma cell line. In 2012, they further discovered that Piezo1 induces mechanically activated cationic currents, and at this point, it could be confirmed that Piezo belongs to MSCs (Kim et al., 2012). However, for several decades, the thorough molecular mechanisms of MSCs in organisms were undeciphered until Adem Pataputian was conferred with the 2021 Nobel Prize in Physiology or Medicine for his unveiling of the existence and function of the mechanoreceptor Piezo ion channel. This significant study has offered novel perspectives on the investigation of mechanoreception in organisms. Piezo channels represent a novel class of MSCs composed of two isoforms, Piezo1 and Piezo2. They consist of non-selective cation channels that promptly and reversibly react to mechanical stimulation, resulting in an extensive inward current and inducing an increase in intracellular calcium ions (Lin et al., 2019; Lewis and Grandl, 2021). Piezo channels are expressed extensively in various tissues and organs, playing crucial roles in both physiological and pathological processes (Lai et al., 2022). These include but are not limited to regulating blood pressure, promoting vascular development, maintaining erythrocyte volume, developing lymphatic vessels, ensuring skeletal homeostasis, and contributing to sensation of touch, pain, and proprioception (Wu et al., 2017a; Gaub and Müller, 2017; Saotome et al., 2018). In recent years, numerous research studies have uncovered the expression, function, and regulation of Piezo channels in the intestines, and their correlation with diseases related to the gut. In this review, we summarize the expression distribution, physiological functions, and pathological roles of Piezo channels in the GI tract, discuss current research advances on Piezo channels in GI disorders, and highlight the potential importance of targeting this family of cation channels for the treatment of GI disorders.

## 2 Overview of Piezo channels

### 2.1 Structure and characteristics of Piezo channels

**FIGURE 1:**
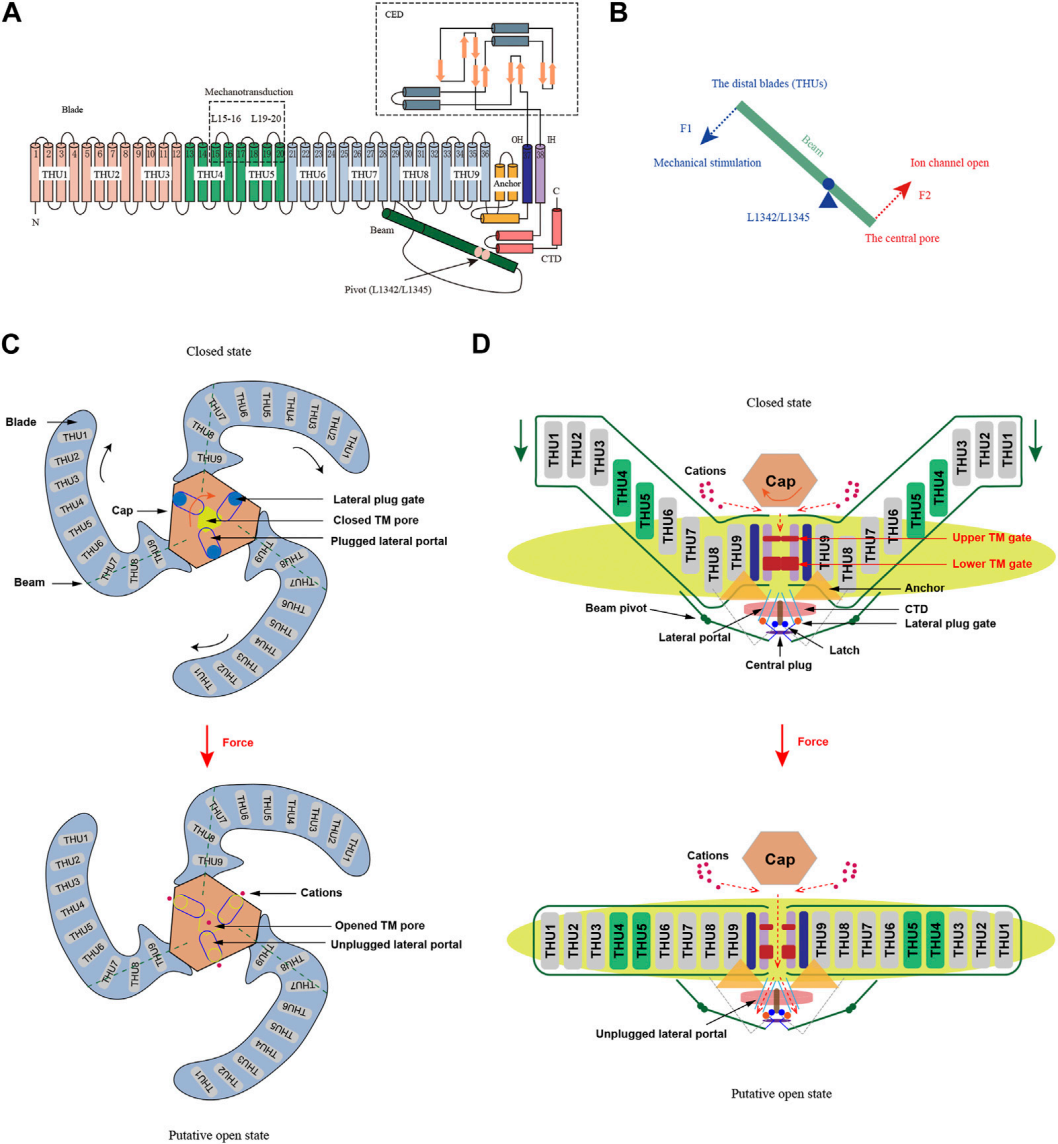
Structure and mechanogating mechanisms of Piezo channels (adapted from Zhao et al., 2018; Li et al., 2022). **(A)** A 38-TM topology structure of Piezo channels. A model showing one subunit with consists of a peripheral blade, a C-terminal structural domain (CTD), a C-terminal extracellular structural domain (CED), an anchor, and an intracellular beam. The extracellular loops EL15-16 and EL19-20 are the key mechanotransduction sites for are crucial for mechanical sensing and transduction, and L1342/L1345 are proposed to form the pivot of the beam. **(B)** Lever-like mechanotransduction model of Piezo channels. The blue and red dashed arrows indicate input and output forces, respectively. **(C)** Extracellular view of Piezo channels. Force-induced clockwise movement of the cap (indicated by the orange arrow) could control the opening of the extracellular fenestration sites and allow cation entry. **(D)** Side view of the Piezo channels. The red dashed arrows indicate closed and open ion conduction pathways, respectively. When the blades flatten under force, the mechanical force transmitted by the beam might unplug the lateral plug gates, opening the intracellular ion-conducting channels and allowing cations to enter.

Over the past decade, the study of Piezo and other MSCs has thrived, with a primary focus on understanding how proteins within cell membranes detect and react to forces. Cryo-electron microscopy (cryo-EM) has rapidly advanced, enabling increasing recognition of Piezo’s distinctive three-blades structure. In 2015, Xiao and colleagues resolved the high-resolution structure of mPiezo1 in mice using cryo-EM and unveiled the crucial components of its mechanosensitivity through biochemical and functional experiments (Ge et al., 2015). Piezo channels comprise a vast array of transmembrane (TM) proteins that constitute 38 TM helices, ultimately forming a homotrimer with three blades. Each subunit consists of a peripheral blade, a C-terminal structural domain (CTD), a C-terminal extracellular structural domain (CED), an anchor, and an intracellular beam (Zhao et al., 2016; Zhao et al., 2018; Tang et al., 2022) (Figure 1). The Piezo channel comprises three functional modules: a mechanosensing module at the N-terminal, a transduction module, and an ion-conducting pore module at the C-terminal (Zhao et al., 2018). The N-terminal mechanosensing module is made up of propeller blades that surround it, each embedded with nine transmembrane helical units (THUs) (Wang and Xiao, 2018). Each THU contains four TM helices, shaped in a curved, non-planar structure that interacts with the lipid bilayer, sensing membrane curvature, or tension changes (Zhao et al., 2018). The mechanotransduction module consists of 90Å long intracellular beams, anchor domain and CTDs (Xiao, 2020). The three CEDs form an extracellular cap structure that sits on top of the helices (OHs) and inner helices (IHs), transmitting mechanical forces from the N-terminus to the C-terminus, causing the opening or closing of the ion-conducting pore (Xiao, 2020; Fang et al., 2021). The central pore module is responsible for ion permeation and selectivity and consists of OHs, IHs, intracellular CTDs, and extracellular CEDs, forming a central pore-forming ion channel that permits the conduction of nonselective cations (Moroni et al., 2018; Wang and Xiao, 2018) (Figure 1). Piezo1 and Piezo2 share only 42% sequence homology and have minor structural differences, including a narrower central pore in Piezo2 than in Piezo1 and an outer cap that wraps more tightly around the central pore in Piezo2 than in Piezo1 (Coste et al., 2010; Jiang et al., 2021a). Despite differences in tissue expression, physiological functions, and biophysical properties between mouse Piezo1 and Piezo2, their overall structure and 38-TM topological organization, along with key structural domains, are strikingly similar. This suggests that Piezo1 and Piezo2 may have similar mechanoregulatory mechanisms to mediate *in vivo* mechanotransduction functions.

### 2.2 Mechanical gating mechanism for Piezo channels

Piezo channels are capable of sensing mechanical stimuli, producing mechanically activated currents, and regulating channel opening through a corresponding mechanical gating mechanism. However, as the Piezo protein’s structure in the open conformation remains unresolved, there is no conclusive experimental evidence regarding the gating mechanism of the Piezo protein. Lever-gating mechanisms have been used to elucidate the function of several mechanically gated ion channels, including TRP cation channel subfamily A member 1 (TRPA1), TRPV4, K2P, and others (Zimova et al., 2018; Zhang et al., 2022; Zhen et al., 2023). These channels share certain similarities, such as an extensive extracellular or intracellular loop and a transmembrane helix attached to the membrane, structures that could act as levers to transmit mechanical stimuli (Xiao, 2020). Structurally, a similar mechanism exists for the Piezo channel, where the THU forms the blade for the mechanosensor and the beam uses the positions of L1342 and L1345 as pivots to form an effective lever-like device that transmits force to the central pore module, thereby opening the lateral channel within the cell (Wang et al., 2018; Zhao et al., 2019) (Figure 1). At the same time, it translates a large conformational change in the distal blade into a relatively small opening in the central pore, ensuring selective cation penetration (Zhao et al., 2019; Xiao, 2020). With respect to the extensive blades, the cap can adopt at least two rotational conformations (Wang et al., 2019). The functional coupling of the cap to the extensive blades has been attributed to electrostatic interactions, indicating a functional correlation between these two regions. In the mechanogating process, the extensive blades are postulated to work in conjunction with the cap by serving as sensors of membrane curvature and tension (Lewis and Grandl, 2020).

A lever-like mechanogating mechanism may enable the piezo to function as an efficient mechanosensor, transforming mechanical stimuli into cationic currents. However, the lever-like mechanogating mechanism is not the sole method for mechanically gated ion channels’ activation. In 2020, the plug-and-latch model for Piezo channels was proposed jointly by research groups led by Xiao and Li (Geng et al., 2020). According to their model, a short peptide known as the plug blocks each intracellular opening of the lateral and central pores of Piezo. The latch, located at the intracellular junction of the blade C-terminus and the CTD structural domain, is also a short peptide. The lateral pore’s three plugs are secured to the central axis of Piezo1 on the cytoplasmic side, connected to the latch by hydrogen bonds between Y1412 and E2537 (Geng et al., 2020; Jiang et al., 2021b). As a result of this transduction mechanism, mechanical stimuli or Piezo chemical activators that act on the peripheral blades can be transmitted to the central axis, leading to the opening of the cytoplasmic gate (Wang et al., 2018). Moreover, the Piezo channels may utilize a dual gating mechanism wherein both the TM gate and lateral plug gate work together in response to various forms of mechanical stimuli (Jiang et al., 2021a; Wang et al., 2022). This mechanism aligns with the diverse and intricate mechanotransduction functions performed by Piezo channels during physiological and pathological processes.

### 2.3 Biophysical properties and pharmacological modulators of Piezo channels

The kinetics of Piezo channels, specifically their activation and inactivation processes, are critical for their role in mechanotransduction. Piezo channels typically exhibit rapid and reversible responses to mechanical stimuli, with an opening time constant measured in milliseconds and an inactivation time constant measured in seconds. Piezo1 channels, one of the most common non-selective cation channels, exhibit high permeability for calcium ions and selective permeability for cations such as Ca^2+^, K^+^, Na^+^, and Mg^2+^(Coste et al., 2015; Ortuste Quiroga et al., 2022). The magnitude of conductance of Piezo1 channels is dependent on their cation-selective permeability (Coste et al., 2010). The mechanism involves lateral membrane tension flattening the Piezo dome, which increases the energy of the membrane-channel system in proportion to the expansion of the projected area of the dome. This energy difference causes the Piezo channels to open, allowing ions to flow through the channel. It is possible to rescue almost all Piezo channels from being inactivated by repeated mechanical stimulation by depolarization causing outward permeation (Li et al., 2017; Moroni et al., 2018).

Certain amino acid residues located in the IH and CTD of the Piezo channel, including L2475, V2476, M2493, and F2494, are central to the inactivation of the channel (Wu et al., 2017b; Zheng et al., 2019). Altering or removing these residues results in changes to the Piezo channel’s inactivation properties (Coste et al., 2015; Zheng et al., 2019). Similarly, the blade structure of Piezo channels can be impacted by certain proteins or small molecules, ultimately influencing mechanosensitivity and ion permeability. Piezo1 and Piezo2 display distinct sensitivities to various mechanical stimuli and exhibit dissimilar inactivation characteristics, which have noteworthy voltage dependence (Coste et al., 2010; Li et al., 2022). To be precise, Piezo2 features faster inactivation kinetics compared to Piezo1, whose inactivation slows down during depolarization (Lewis et al., 2017). Understanding the inactivation kinetics of Piezo channels is therefore critical for understanding their role in mechanotransduction and their potential as therapeutic targets for various diseases.

Several molecules and drugs have been discovered to impact the activity of Piezo channels, including agonists and blockers. Piezo channels can be non-specifically blocked by small molecules like ruthenium red and gadolinium ions (Coste et al., 2012). Peptide toxins like GsMTx4 can inhibit Piezo channels non-specifically by being inserted into the lipid bilayer to reduce membrane tension (Gnanasambandam et al., 2017). Piezo channels activity can be inhibited by various proteins, such as SERCA2. This calcium ion transport pump is located in the endoplasmic reticulum membrane and inhibits Piezo1 channel activity by reducing the effective coupling between the mechanotransduction-module and the pore-module of Piezo1 channel (Zhang et al., 2017).

The presently identified agonists for Piezo1 are Yoda1 and Jedi1/2. They reduce the mechanical threshold and prolong the inactivation time of Piezo1, as well as increase its mechanosensitivity (Lacroix et al., 2018; Botello-Smith et al., 2019). They bind to the blade of Piezo1, promoting blade movement and opening the channel (Syeda et al., 2015). Yoda1 and Jedi1/2 have distinct action sites, resulting in varying activation effects. And there is currently no recognized agonist for Piezo2. In addition, some endogenous compounds, including fecal ssRNA and ceramides, may serve as natural regulators or ligands of Piezo1 and modulate its functions (Shi et al., 2020; Sugisawa et al., 2020). GsMTx4, a positively charged amphipathic molecule, can embed into lipid bilayers and suppress Piezo1 currents (Copp et al., 2016; Suchyna, 2017). A Yoda1 analogue, known as Dooku1, has been employed in different cell types to counteract the effects induced by Yoda1. Dooku1 can reversibly hinder Yoda1-induced effects but is incapable of activating Piezo channels (Hatem et al., 2023). To date, the molecular signaling mechanism and the signaling interaction of Piezo channels have not been studied in depth, and the research of drugs targeting Piezo channels has just begun, especially the specific action of Piezo2 channel proteins is still blank. As a result, the thorough investigation of the pharmacological properties of piezo will lead to the development of a new phase of corresponding target drugs.

### 2.4 Biological functions of Piezo channels

Mechanical forces are present throughout biological growth and development, and MSCs play a crucial role in osmotic pressure regulation, cell growth and proliferation, morphogenesis, proprioception, and locomotion due to their distinctive structural and physiological features (Kefauver et al., 2020; Inman and Smutny, 2021). Piezo channel proteins are widely expressed in a multitude of cells and are involved in a variety of physiological and pathological processes, and current research highlights the potential of piezo as a novel drug target for disease intervention in bone, cardiovascular system, innate immunity, and human cancers (Tang et al., 2022). Piezo maintains normal cellular morphology and is closely related to metabolism and cell motility. Cahalan et al. (2015) found that reduced expression or knockdown of Piezo1 resulted in decreased influx of calcium ions in erythrocytes and that Piezo1 activators, such as Yoda1, induced erythrocyte dehydration similar to hereditary xerocytosis (HX). Thus, Piezo1 plays a critical role in maintaining cellular homeostasis by responding to mechanical force signals.

Cell differentiation and cell fate decisions are regulated by various external signals, including mechanical force signals, and the piezo ion channel-related cell differentiation process has received extensive attention. In 2014, Tombola’s research group first discovered that Piezo1 can promote neural stem cell differentiation into neurons by inducing Ca^2+^ influx, while MSC-specific inhibitors such as GsMTx4 can suppress neuronal formation (Pathak et al., 2014). Studies have shown that Piezo1 is a key mechanosensor required for bone development and osteoblast differentiation. Loss of Piezo1 in bone marrow mesenchymal stromal cells (BMSCs) or osteoprogenitors results in impaired osteoblast differentiation and spontaneous bone fractures in newborn mice (Sugimoto et al., 2017; Zhou et al., 2020). Piezo also plays an important role in the formation and development of tissues and organs. For example, Piezo1 participates in the development and regulation of the circulatory system by sensing blood flow and mechanical pressure. When the blood flow increases, Piezo1 opens more, the cell membrane depolarizes more, and the blood vessels contract. Knockout of Piezo1 causes defects in embryonic endothelial vascular development and angiogenesis, leading to embryonic lethality (Li et al., 2014; Ranade et al., 2014). In addition, Piezo1 has been shown to specifically inhibit Treg cells to alleviate experimental autoimmune neuritis (Jairaman et al., 2021). Notably, several studies have shown that the expression of Piezo1 is associated with the clinical features of cancer patients, and high levels of Piezo1 are negatively correlated with the overall survival of tumor patients, making Piezo1 a novel biomarker for the diagnosis and prognosis of various human cancers (Dombroski et al., 2021; Yu and Liao, 2021). In the intestine, Piezo1 significantly contribute to the mechanical regulation of the intestinal epithelium by shaping stem cell zones (SCZs) in organoids. Their involvement in fission events within SCZs influences organoid patterning and morphogenesis, driving acute stem cell differentiation and inducing a stretch-responsive cell state in the intestinal epithelium, as reflected in the expression levels of stretch signature marker genes (Tallapragada et al., 2021). Piezo1 mechanotransduction, activated by the inflation of closed-lumen organoid crypts, is instrumental in driving the fragmentation of the stem cell niche within intestinal crypts. This process is further evidenced by crypt fission triggered during the deflation of the crypt, highlighting the integral role of Piezo1 channels in this mechanosensitive phenomenon (Pérez-González et al., 2022).

## 3 Expression and distribution of Piezo channels in the intestine

**FIGURE 2:**
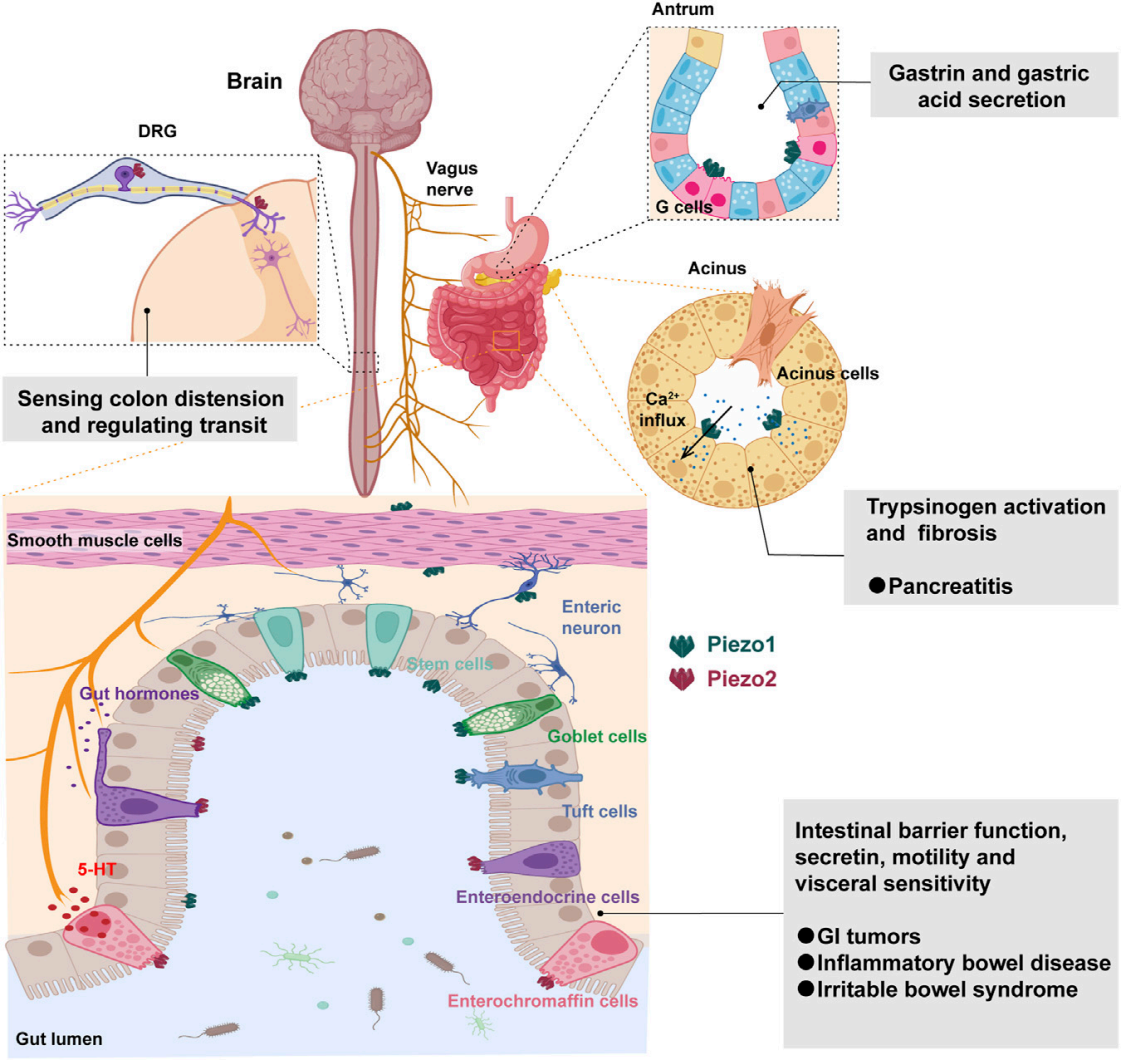
Mechanosensing functions and roles of Piezo channels in the gastrointestinal tract. Piezo2 in the dorsal root ganglia (DRG) senses colon distension and regulates intestinal transit. Piezo1 facilitates the release of gastrin and gastric acid from G cells in the antrum. The activation of Piezo1 in pancreatic acinar cells induces sustained intracellular calcium influx, triggering pancreatitis and fibrosis. Piezo channels have a broad expression distribution in the intestinal tract, including epithelial cells, smooth muscle cells, neurons and enteroendocrine cells. Piezo1 is predominantly expressed in stem cells, goblet cells, and tuft cells, while Piezo2 is primarily expressed in enteroendocrine cells. They play crucial roles in intestinal barrier function, secretion, motility, and visceral sensitivity, implicating their involvement in GI tumors, inflammatory bowel diseases, and irritable bowel syndrome.

During physiological processes such as digestion and absorption, the intestine is constantly exposed to various forms of mechanical stimuli, such as the osmotic pressure of food and GI motility. Mechanical force is essential to maintain normal function of GI epithelial cells (Mercado-Perez and Beyder, 2022). Piezo channels have a broad expression distribution in the GI tract, including epithelial cells, smooth muscle cells, neurons and enteroendocrine cells (EECs) (Joshi et al., 2021; Yang et al., 2022). Piezo1 and Piezo2 have some overlap in their expression patterns in the GI tract, but also some differences, reflecting their different functions in the intestine. Piezo1 is mainly expressed in the epithelial cells, smooth muscle cells and enteric neurons of the GI tract, while Piezo2 is mainly expressed in the EECs, with low expression in the enteric neurons but high expression in the dorsal root ganglia (DRG), thereby regulating intestinal mechanosensation (Bai et al., 2017; Lang et al., 2018; Servin-Vences et al., 2023) (Figure 2). The expression level and distribution pattern of Piezo channels may be related to different types of mechanical stimuli and intensities, such that Piezo1 is mainly distributed at the bottom of the ileal and colonic crypts, while Piezo2 is distributed along the ileal crypt-villus axis but mainly in the lower half of the colonic crypts (Tallapragada et al., 2021; Jones et al., 2022). The expression of Piezo channels may also be influenced by factors such as age, gender, nutritional status, hormone levels, inflammatory status, etc., (Ranade et al., 2014; Zhao et al., 2018; Liu et al., 2023; Migulina et al., 2023). For example, Piezo1 is more highly expressed in the small intestinal epithelial cells of aged rats (Sugisawa et al., 2020), whereas Piezo2 levels in the intestine decrease with age (Jones et al., 2022), and Piezo2 is more highly expressed in EC cells (Wang et al., 2017).

It has been found that Piezo1 and Piezo2 are both expressed in EECs and involved in the release of 5-hydroxytryptamine (5-HT) (Linan-Rico et al., 2016; Sugisawa et al., 2020; Treichel et al., 2022). Piezo1 may be involved in mucus secretion in goblet cells and chemosensation in tuft cells (Bai et al., 2017; Lang et al., 2018; Xu et al., 2023), but more importantly, when intestinal epithelial cells are overcrowded, Piezo1 channels are activated, triggering the extrusion of live cells, thereby achieving the purpose of controlling the number of epithelial cells (Yang et al., 2014; Pérez-González et al., 2022). Mazzuoli-Weber et al. (2019) found that Piezo1 was expressed in the neuronal cell bodies, myenteric plexus and submucosal plexus of the guinea pig, mouse and human GI tract by immunohistochemistry. In contrast, Piezo2 expression was very rare in enteric neuronal cell bodies and present in a few axons. Currently, it is known that Piezo channels can be expressed in airway smooth muscle and vascular smooth muscle and may play a role in tension regulation, vasodilation, etc. In comparison, little is known about whether piezo proteins in GI smooth muscle cells and ICCs are involved in intestinal motility. It is noteworthy that beyond the gastrointestinal tract, the sensitivity of the pancreas to mechanical pressure is closely related to the functionality of the Piezo channels. Piezo1 is expressed in pancreatic acinar cells and plays a crucial role in mediating pressure-induced pancreatitis by facilitating calcium influx and causing pancreatic injury (Romac et al., 2018; Zhu et al., 2022b). Additionally, Piezo1 expression in pancreatic stellate cells contributes to pancreatic fibrosis through increased expression of TGF-β1, fibronectin, and type I collagen, thereby implicating Piezo1 activation in the development of pressure-induced chronic pancreatitis and fibrosis (Swain et al., 2022) (Figure 2).

## 4 Physiological roles of Piezo channels in the GI tract

### 4.1 Role of Piezo channels in gastric acid secretion

Gastric acid secretion is a complex process regulated by the nervous, endocrine and parasympathetic systems. Among them, mechanical stimulation is an important regulatory factor, which can activate the vagus nerve through receptors, promoting the release of histamine and prostaglandin by parietal cells, thus stimulating the secretion of hydrochloric acid by parietal cells (Page et al., 2002; Magris et al., 2020). Piezo channels, specifically Piezo1, have been shown to play a role in gastric acid secretion. G cells in the antrum of the stomach that produce gastrin, a hormone involved in the control of gastric activities, express Piezo1 channels. These channels are mainly located in the basolateral part of the G cells, which is the release site for gastrin secretion (Lang et al., 2018). This finding suggests that Piezo1 may sense the pressure changes of food or liquid on the gastric wall and stimulate G cells to secrete gastrin, thereby increasing gastric acid secretion. While the specific role of Piezo channels in gastric acid secretion is still under investigation, their presence in the GI tract suggests their involvement in mechanosensation and regulation of GI function (Alcaino et al., 2017a).

### 4.2 Role of Piezo channels in intestinal barrier function

Intestinal barrier function refers to the isolation and protective role of the intestinal epithelial layer from the external environment, including physical barriers, biochemical barriers and immune barriers (Zhang et al., 2023). Mechanical stimulation plays a critical role in intestinal barrier function. It can affect the junctions between epithelial cells, regulate the thickness of the mucus layer and influence the activity of immune cells. The secretion of mucus by goblet cells and the specific tight junction (TJ) architecture of the intestinal epithelium are important mechanisms involved in the protection of the intestinal barrier (Gustafsson and Johansson, 2022). Recently, Xu et al. (2021) confirmed for the first time that mechanical stimulation can activate Piezo1 to promote mucin2 expression in goblet cells. Similarly, a study reported that mice exposed to water avoidance stress (WAS) showed decreased mucus layer thickness, increased mucus permeability, decreased goblet cell number, and decreased Piezo1 expression (Xu et al., 2023). Notably, the Piezo1 agonist Yoda1 can alleviate mucus barrier damage in mice exposed to WAS by inhibiting H3K9me3 modification and promoting mucin2 expression (Xu et al., 2023). Liu et al. (2022) also found that the altered mucus layer and dysbiosis in Piezo1-deficient mice can result in an impaired gut microenvironment, increased encounters with microbial antigens, and a mild pro-inflammatory state. These findings suggest that Piezo1 in goblet cells plays a critical role in maintaining the balance of the fecal microbiota and intestinal homeostasis. On the other hand, Piezo1 can negatively regulate epithelial barrier function by affecting the expression of Claudin-1, a key protein involved in TJs (Jiang et al., 2021b). Activation of Piezo1 can induce epithelial dysfunction. Therefore, Piezo1 plays a role in maintaining intestinal barrier function by regulating TJs and goblet cell function in response to mechanical stimulation.

### 4.3 Role of Piezo channels in intestinal secretin release

Enteric secretin refers to a class of hormones secreted by intestinal endocrine cells, such as glucagon-like peptide-1 (GLP-1), cholecystokinin (CCK), and vasoactive enteropeptide (VIP). These hormones are produced by EECs, including L-cells, K-cells, and I-cells found in the ileum and colon (Bany Bakar et al., 2023). The release of intestinal secretin is regulated by several factors, among which mechanical stimulation is an important regulatory factor that can activate neurons or act directly on endocrine cells, thereby stimulating or inhibiting the release of intestinal secretin (Treichel et al., 2018; Joshi et al., 2021). Previously, Piezo2, a tactile sensing protein, was found not only in the fingers but also in the intestine. More interestingly, it was recently discovered that Piezo2 appears to play an important role in the intestinal secretin release process. The presence of Piezo2 in EC cells was confirmed using multiple mouse models and super-resolution microscopy (Alcaino et al., 2018). Activation of Piezo2 in EC cells leads to a rapid inward ionic current and an increase in intracellular calcium, resulting in mechanosensitive serotonin release (Wang et al., 2017; Dickson, 2018). Inhibition of Piezo2 by drugs or molecular knockdown reduces mechanosensitive currents, serotonin release and downstream physiological effects (Alcaino et al., 2017b; Alcaino et al., 2018). Thus, Piezo2 is critical for EC cells mechanosensitivity and 5-HT release.

### 4.4 Role of Piezo channels in intestinal motility

Intestinal motility is a periodic contraction and relaxation activity controlled by smooth muscle cells and neurons that is essential to propel food and waste in the intestine (Kumral and Zfass, 2018). Intestinal motility is regulated by various factors such as neurons, smooth muscle cells, interstitial cells, neurotransmitters, hormones, etc. Of course, some studies have found that Piezo1 and Piezo2 are also involved in the regulation of intestinal motility. A recent study published in Gastroenterology has shown that EECs require the Piezo2 receptor to sense the size of luminal contents and modulate motility throughout the intestinal tract (Treichel et al., 2022). As mentioned above, activation of Piezo2 in EECs allows them to transmit sensory information to surrounding enteric neurons responsible for motility and secretion, thus contributing to the coordination of intestinal functions (Alcaino et al., 2018). Further, Piezo2 activation in EECs leads to the release of signaling molecules such as serotonin, substance P, and peptide YY. These molecules may have additional effects on intestinal motility and merit further investigation (Najjar and Margolis, 2022; Treichel et al., 2022). Although the role of Piezo1 in GI smooth muscle cells is still unclear, its biophysical properties indicate that it can sense mechanical stimulation from food or fluid, thereby activating calcium signaling and contractile proteins and promoting smooth muscle cell contraction. Consequently, piezo may be a novel mechanosensitive factor that regulates intestinal motility.

## 5 Expression and function of Piezo channels in GI diseases

### 5.1 GI tumors

Piezo channels are involved in translating mechanical stress into Ca^2+^-dependent signals and contribute to altered calcium signaling in cancer cells (De Felice and Alaimo, 2020), but the exact role of Piezo channels in cancer is still not fully understood and may vary depending on the specific cancer type. For instance, Piezo channels have been found to be implicated in the proliferation and metastasis of GI tumors, particularly colon and gastric cancer (Sun et al., 2020; Wang et al., 2021). High levels of Piezo2 expression have been observed in colon cancer tissues and are associated with poor prognosis (Shang et al., 2023). Knockdown of Piezo2 attenuates the proliferation, migration and invasion of SW480 cells through the SLIT2/ROBO1/VEGFC pathway (Shang et al., 2023). Mechanistically, Piezo1 activation leads to the activation of downstream signaling pathways, such as the PI3K/Akt and ERK pathways, which are known to regulate cell proliferation (Yu and Liao, 2021). Inhibition of Piezo1 has been shown to suppress cancer cell proliferation, suggesting that targeting Piezo1 may have therapeutic potential in inhibiting tumor growth.

Increased expression of Piezo1 was observed in gastric cancer cell lines and primary samples, and this upregulation was associated with poor disease-specific survival. Knockdown of Piezo1 led to inhibitory effects on cell proliferation and invasion, as well as inhibiting xenograft formation. Moreover, Piezo1 knockdown enhanced the sensitivity of Cisplatin or 5-FU treatment (Zhang et al., 2018). Likewise, Piezo1 is highly expressed in gastric cancer (GC) tissues with omentum metastasis and metastatic lymph node tissues, suggesting its role in GC omentum metastasis (Wang et al., 2021). In GC cells, Piezo1 facilitates cell migration and controls calpain 1/2 expression by regulating HIF-1α. Knockdown of Piezo1 inhibits peritoneal metastasis of GC cells, blocks the epithelial-mesenchymal transition (EMT) process, and inhibits angiogenesis *in vivo* (Wang et al., 2021). Additionally, Piezo1 is considered a potential tumor-promoting marker and its inhibition may inhibit the progression of pancreatic ductal adenocarcinoma (Zhu et al., 2022a). Since Piezo1 activation accelerates tumor growth and modulates the tumor microenvironment in pancreatic cancer, these findings suggest that Piezo channels, particularly Piezo1, have the potential to be novel therapeutic targets for drug design and treatment of GI tumors.

### 5.2 Pancreatitis

Pancreatitis, a prevalent digestive disorder, is typified by the inflammatory and necrotic processes within the pancreatic tissue, resulting in impaired pancreatic function and the onset of severe complications (Yu et al., 2022). The pancreas is sensitive to mechanical injury, and manipulation during surgery or blunt abdominal trauma are common causes of pancreatitis. It was once thought that gallstone impaction caused pancreatitis, but later studies showed that back pressure from pancreatic duct obstruction may also be a cause. Increased pressure within the pancreas, such as from overfilling the pancreatic duct during diagnostic procedures, can trigger acute pancreatitis (Malgras et al., 2011; Romac et al., 2018). However, how the pancreas perceives and responds to mechanical stimuli and how mechanical stimuli trigger pancreatitis remains an unanswered question. Whether the expression and function of pancreatic piezo channels is related to the onset and progression of pancreatitis is a question worth exploring.

Recent studies have shown that Piezo1 is expressed in pancreatic acinar cells and responds to static pressure, shear stress and membrane stretch in the pancreatic duct, while its activation leads to calcium influx and subsequent pancreatic injury (Romac et al., 2018). Excessive cytoplasmic calcium signals are toxic to pancreatic acinar cells and disrupt normal intracellular calcium signaling, leading to abnormal fusion of zymogen and lysosomal granules and premature activation of enzymes (Feng et al., 2019). Infusion of the Piezo1 agonist Yoda1 through the pancreatic duct was shown to induce all parameters of pancreatitis in mice, including pancreatic edema, hemorrhage, necrosis and inflammatory cell infiltration, as well as elevated blood amylase and myeloperoxidase. In contrast, the administration of GsMTx4 or the genetic deletion of Piezo1 in acinar cells protected mice against pressure-induced pancreatitis. This finding further supports the role of Piezo1 in the development of pancreatitis (Romac et al., 2018). Strikingly, a series of *in vitro* and *in vivo* studies have shown that sustained elevation of intracellular calcium, leading to enzyme activation and cell death, is caused only by high and prolonged pressure applied to the pancreas (Swain et al., 2020). However, sustained elevation in intracellular calcium is necessary for pancreatitis, and this study demonstrates that Piezo1 activation triggers the opening of the TRPV4 channel, which leads to sustained elevation in intracellular calcium and the development of pancreatitis. Moreover, further studies demonstrate that elevated pancreatic pressure induces Piezo1 activation, which triggers TRPV4 opening and calcium influx, thereby activating stellate cells and initiating chronic pancreatitis and fibrosis (Swain et al., 2022). Overall, piezo channel inhibition may be a promising intervention for pancreatitis. However, further studies are needed to elucidate the precise role and molecular mechanisms of piezo channels in pancreatitis. Piezo channel research in pancreatitis provides a novel insight and opportunity to explore the mechanobiology of the pancreas and to discover new therapeutic strategies for pancreatitis.

### 5.3 Inflammatory bowel disease

Inflammatory bowel disease (IBD) is a chronic inflammatory disease of the intestine, including Crohn’s disease (CD) and ulcerative colitis, whose pathogenesis is still unclear but may be related to genetic, environmental and microbial factors (Shan et al., 2022). Most recently, there has been increasing evidence that MSCs play an important regulatory role in the pathogenesis of IBD. On the one hand, MSCs can sense physical changes in the intestine, like motility, tension, pressure and shear force, and regulate intestinal secretion, absorption, barrier and immune functions (D'Alessio et al., 2022; Swain and Liddle, 2023). On the other hand, MSCs can also be affected by inflammatory mediators, which can alter their expression and activity and hence influence the intestinal inflammatory response and fibrosis process (Sugisawa et al., 2020; Waclawiková et al., 2022). For example, TRPA1 and TRPV1 are highly expressed in intestinal epithelial cells and stenotic tissue samples from patients with IBD, affect the progression of colitis and fibrosis in mice, and contribute to the development of nociceptive hypersensitivity and inflammation in the intestinal mucosa (Rizopoulos et al., 2018; Csekő et al., 2019; Inoue et al., 2019). In contrast, there are few studies on the biological effects of Piezo channel in IBD. Liu et al. (2023) found that Piezo1 was significantly expressed in the ileum of patients with CD and positively correlated with disease activity and inflammatory markers. Activation of Piezo1 in intestinal epithelial cells leads to calcium influx, resulting in mitochondrial dysfunction and activation of the NLRP3 inflammasome (Liu et al., 2023). The findings suggest that targeting Piezo1 or modulating calcium signaling pathways may be potential therapeutic strategies for CD. Overall, the mechanisms by which Piezo may affect IBD remain largely unknown, but may be related to its role in calcium influx and mitochondrial dysfunction, Clearly, the effect of Piezo inhibition on other aspects of intestinal inflammation, such as immune cell activation and cytokine production, should be investigated to gain a comprehensive understanding of its role in the inflammatory process.

### 5.4 Irritable bowel syndrome

Irritable bowel syndrome (IBS) is a chronic functional intestinal disorder characterized by abdominal pain or discomfort accompanied by changes in bowel habits or stool characteristics (Barbara et al., 2016). The pathogenesis of IBS is not fully understood, but GI motility and visceral sensory dysfunction are thought to be the main pathophysiological bases of IBS. The 5-hydroxytryptamine (5-HT) signaling pathway is the most prominent in IBS research. 5-HT can regulate intestinal motility, GI secretion and gut sensation, and is also an important neurotransmitter involved in the brain-gut axis. EC cells release 5-HT when stimulated by physical and chemical stimuli in the intestinal lumen, which can lower the threshold of visceral sensation and stimulate phasic colonic contraction by increasing the release of acetylcholine (ACh) at the nerve effector junction (Gunn et al., 2019; Jones et al., 2020). Piezo2 is known to play a role in mechanically induced pain syndromes and is essential for visceral hypersensitivity. IBS patients all experience 5-HT-induced nociceptive responses (Keszthelyi et al., 2015), and Piezo2 channels can induce 5-HT release when activated by mechanical stimuli (Alcaino et al., 2018). Therefore, Piezo2 in the colon is closely related to 5-HT (Guo et al., 2022).

In a study using rats as a model organism, Piezo2 knockdown in dorsal root ganglia attenuated visceral sensation to innocuous and noxious stimuli (Yang et al., 2016). Xie et al. (2023) found that Piezo2 channels in TRPV1 lineage neurons are key mediators of visceral mechanosensitivity and nociception under normal conditions. Ablation of Piezo2 channels reduces visceral afferent action potential firing and visceromotor responses in both physiological states and mouse models of IBS and partial colonic obstruction. Interestingly, in GI transit, lack of Piezo2 in sensory neurons leads to delayed evacuation and diarrhea-like behavior, possibly due to failure to resorb water caused by reduced transit times (Servin-Vences et al., 2023). Collectively, these findings indicate the value of exploring the potential of Piezo2 as a biomarker for IBS, providing a potential therapeutic target for the treatment of visceral pain and abnormal GI motility.

## 6 Conclusion

In this comprehensive review, we have meticulously examined the expression, distribution, physiological functions, and pathological roles of Piezo channels within the gastrointestinal (GI) tract. Our analysis has illuminated the intricate involvement of Piezo channels in pivotal physiological processes, such as gastric acid secretion, intestinal barrier function, secretin release, and motility. Furthermore, we explored their significant contributions to GI-related diseases, including tumors, pancreatitis, inflammatory bowel disease, and irritable bowel syndrome (Figure 2). The revelations from our synthesis underscore the profound impact of Piezo channels on the mechanobiology of the GI tract. While our review has provided valuable insights into their roles, it is imperative to acknowledge the existing gaps in our understanding. Notably, the mechanical gating mechanism, pharmacological properties, signal transduction pathways, and subcellular localization of Piezo channels in the intestinal tract remain areas of uncertainty, necessitating further experimental validation and theoretical elucidation. Moreover, the potential formation of complexes or networks between GI Piezo channels and other mechanosensitive ion channels or proteins presents an intriguing avenue for future research. The collaborative participation of these elements in intestinal mechanosensation and regulation poses an exciting prospect for uncovering novel aspects of GI physiology.

As we contemplate the future trajectory of Piezo channel research in the GI tract, it becomes evident that the ongoing exploration holds promise for breakthroughs in the prevention and treatment of intestinal diseases. The unanswered questions and uncharted territories surrounding Piezo channels beckon researchers to delve deeper into their complexities. We anticipate that continued investigations will not only address the existing gaps in knowledge but also unveil new dimensions, ultimately contributing to innovative approaches in diagnosing and treating GI-related disorders.
